# Insights into Isolation and Purification Strategies of Egg Allergens

**DOI:** 10.3390/foods14111944

**Published:** 2025-05-29

**Authors:** Nikolina Sibincic, Ivana Prodic, Danijela Apostolovic, Christine Y. Y. Wai, Agnes S. Y. Leung, Marija Stojadinovic

**Affiliations:** 1 Innovative Centre, Faculty of Chemistry, University of Belgrade, 11158 Belgrade, Serbia; nsibincic@chem.bg.ac.rs; 2 Department of Research and Development, Institute of Virology, Vaccines and Sera “Torlak”, 11221 Belgrade, Serbia; iprodic@torlak.rs; 3 Division of Immunology and Respiratory Medicine, Department of Medicine Solna, Karolinska Institutet, Karolinska University Hospital, 171 77 Stockholm, Sweden; 4 Center for Molecular Medicine, Karolinska Institutet, 171 77 Stockholm, Sweden; 5 Department of Paediatrics, Faculty of Medicine, Prince of Wales Hospital, The Chinese University of Hong Kong, Hong Kong, China; christineyywai@cuhk.edu.hk (C.Y.Y.W.); agnes.syl@cuhk.edu.hk (A.S.Y.L.); 6 Hong Kong Hub of Pediatric Excellence (HOPE), The Chinese University of Hong Kong, Hong Kong, China; 7 Department of Biochemistry, Center of Excellence for Molecular Food Sciences, Faculty of Chemistry, University of Belgrade, 11158 Belgrade, Serbia

**Keywords:** allergens, egg allergy, egg white, egg yolk, extraction, purification

## Abstract

Eggs are a great source of protein in the human diet. They are consumed in tens of millions of tons globally per year. In addition, egg proteins, which are known food allergens, are included in many food products due to their excellent techno-functional properties. Hen’s eggs are the most consumed, but other edible avian eggs are occasionally used as gourmet ingredients or delicacies. With a high presence in the food market, the risk of accidental exposure to egg allergens is high. Hen egg allergy ranks among the top three food allergens in infants and young children. The complex structure and similar physicochemical properties of egg proteins limit their separation and purification, making further research challenging. Egg composition is influenced by age, disease, medicine, and environmental stress, and the target protein is often present in negligible amounts or polymorphic forms. To investigate the immunoreactivity of proteins from eggs of different bird species, it is necessary to consistently and quantitatively extract and purify proteins while avoiding harsh conditions. The conformational shape of allergens is impacted by denaturation, which can remove or expose IgE-binding epitopes and change the allergenic potential of proteins. This review presents findings from a literature survey on the isolation and purification strategies utilized for egg allergens from culinary-relevant bird eggs.

## 1. Introduction

Eggs are classified as nutrient-rich food because they contain high-quality protein, unsaturated fats, vitamins, minerals, and other nutrients with recognized health benefits [1]. Eggs are versatile and used in diverse culinary applications. Moreover, they serve as a cost-effective protein source, particularly beneficial for individuals facing food insecurity [2]. As a result, food science and technology have placed significant importance on eggs and egg products, implementing methods for the extraction and separation of egg components. However, hen egg (HE) allergy is among the top three food allergies in infants and children [3], and its frequency has been increasing in recent years [4]. In the first two years of life, the incidence of HE allergy in Europe is believed to be around 1.23% [5]. While egg allergies are common in early childhood, most are outgrown over time, leading to a significantly lower prevalence among adults [6]. The most serious consequences of HE allergy include anaphylactic reactions [7], and the potential to trigger non-IgE-mediated food allergies, such as food protein-induced enterocolitis syndrome (FPIES) [8,9]. Moreover, food allergies, including HE allergy, decrease the quality of life in affected individuals by restricting children’s and parents’ social activities. Bullying, melancholy, anxiety, attention/deficit hyperactivity disorder, and increased healthcare expenditures are also related to food allergies [9]. Given that HEs are rich in nutrients, the complete avoidance of HE consumption in the first years of life as an act of preventing severe consequences may result in a nutritional imbalance [10]. Hence, strategies for the partial inclusion of eggs, such as low doses of heated HEs, have been implemented to raise the tolerance threshold for allergic children and reduce the risk of accidental reactions to HE [11,12].

To our knowledge, the literature contains only a limited number of studies addressing the mono- and cross-reactivity of egg proteins from avian species other than hens, such as duck [13], goose [14], quail [15], ostrich [16], turkey [17], pheasant [18], seagull [19], guinea fowl [20], pigeon [21], partridge [21], and emu [22] eggs. Although allergens in HE white are cross-reactive with the different turkey, duck, goose, and seagull egg whites, the degree of cross-reactivity varies. A correlation was observed between the degree of immunological cross-reactivity and the degree of amino acid sequence similarity [23]. Sensitization to avian egg proteins without hen egg sensitization has also been described; an adult patient who had no sensitivity to hen eggs showed an IgE-mediated allergic response to duck and goose egg whites [23]. In another study, it was found that the amino acid differences between quail and hen egg ovomucoid are mainly in the IgE epitopes, being found in eight out of nine epitopes [15]. These variations may affect differences in the allergenicity of the same proteins across the two species. A case has been reported of a child who experienced anaphylaxis after consuming a raw quail egg, despite having no prior allergic reactions to boiled quail or chicken eggs. A prick-by-prick test (PPT) was performed for quail’s egg, which was positive, but this test was negative for HE. IgE binding to quail ovotransferrin was detected in an immunoblot without any similar bands in HE [24]. There was also a report describing a HE-allergic child experiencing anaphylaxis at the first contact with quail egg, with a strong correlation between HE and quail egg PPT positivity [25].

Many proteins found in the eggs of different birds other than hens are not well characterized, and some of them are still unknown. Insufficient research can be attributed to the eggs’ complex structure and similar physicochemical properties of the proteins, which limit their separation and purification. Moreover, the concentration of each protein in the native albumen and yolk varies greatly, as egg composition is influenced by age, disease, medicine, and environmental stress [26]. Additionally, the target protein is often present in negligible amounts or exists in various polymorphic forms (or genetic variants) that frequently only differ in their core sequence by one or two amino acids, which is another obstacle. In addition, after being synthesized, many egg proteins undergo posttranslational modifications [27]. Considering all the above, the process of separating and purifying egg proteins is not straightforward, thus limiting further research. Therefore, we prepared a literature overview of the methodologies and their effectiveness in extracting and purifying egg allergens, broadening our inquiry to include other bird species besides hens. Identifying, quantifying, and assessing the allergic sensitization potency of egg allergens across species is important as the consumption of avian/hen eggs has increased in the last decade, as has the exposure to egg allergens. 

## 2. Methodology

A comprehensive literature review was conducted across the PubMed, Scopus, and Google Search databases. The review included publications from the time these platforms became available up to the present, with particular emphasis on studies published in the past few years to capture recent advancements in the field. Only peer-reviewed articles published in English-language scientific journals were included in the review. Additionally, Chinese research platforms were consulted for egg allergy prevalence data. To obtain more detailed information on individual allergens in hen’s egg and the eggs of other avian species, the allergen database maintained by the World Health Organization and the International Union of Immunological Societies (WHO/IUIS) Allergen Nomenclature Sub-Committee: https://www.allergen.org/ (accessed on 13 March 2025) was consulted.

The search strategy applied to these databases involved the use of specific search terms, including “egg white OR yolk”, “hen OR avian egg allergy”, “egg allergens”, “IgE-mediated egg allergy”, “egg sensitization”, “egg allergen isolation OR extraction OR separation OR purification methods OR techniques”, “egg allergen characterization”, as well as various combinations and word variations of these terms.

Studies were selected based on the following inclusion criteria: (a) methods of isolation and purification of egg allergens are described, (b) the application of a co-purification strategy for hen egg white allergens, (c) the characterization of isolated proteins according to purity, yield, structural analysis or IgE-binding activity. Conference abstracts and preprints were excluded. After duplicates were removed, the titles and abstracts of the identified studies were independently screened by the authors. Studies that were not relevant to the research topic were excluded during this stage. Full-text articles were subsequently reviewed by the authors to determine their suitability for inclusion.

## 3. Egg Production and Consumption Across the Globe

**Figure 1:**
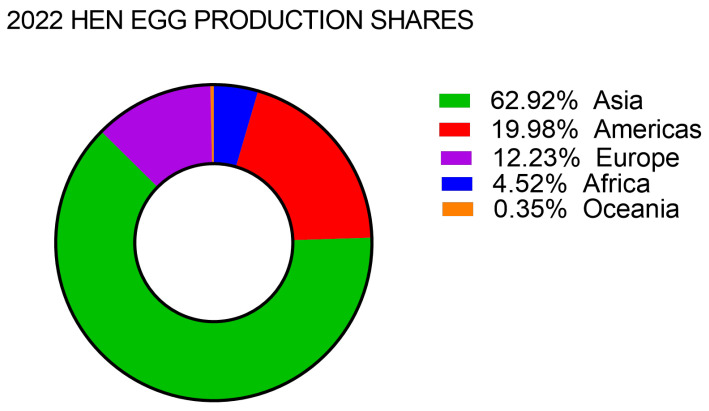
Global fresh hen egg production in 2022. Retrieved from the FAO Crops and livestock production data set, available at http://www.fao.org/faostat (accessed on 3 March 2025).

Global egg production has grown steadily over the last decades. The most recent data on global egg production from FAO (Food and Agriculture Organization of the United Nations) estimate that a total of 8.70 × 10^7^ tons (t) of HEs were produced in the world in 2022, with 6.42 × 10^7^ t in 2010, and 5.12 × 10^7^ t in 2000 (source: FAOSTAT Crops and livestock production data, available at http://www.fao.org/faostat (accessed on 3 and 17 March 2025). Asia accounted for almost 63% of total egg production in 2022, followed by the Americas at 20% and Europe at 12% of production shares (Figure 1).

We accessed the FAOSTAT Supply Utilization Accounts from the last decade to calculate the global percent increase in the daily per capita supply of fresh HEs (Table 1). There has been an average 21% increase in the daily supply of fresh HEs in the world from 2010 to 2020. The most significant change in egg supply from 2010 was seen in the regions of the world with lower egg consumption. Micronesia had the highest rise in HE supply at 308%, followed by Southeast (119%), South (62%), and Central Asia (66%).

## 4. Egg Allergy Prevalence

Egg allergy is one of the most prevalent food allergies, particularly among children. Globally, the prevalence of HE allergy varies, and demographic factors, including race and ethnicity, or socioeconomic factors, influence the prevalence of HE allergy. Recent studies have provided updated insights into the prevalence and demographic variations in HE allergy. In the United States, a comprehensive national survey across diverse racial groups conducted between October 2015 and September 2016 assessed parent reported HE allergy prevalence among children. The findings indicated that approximately 0.9% of all children and 1.3% of children under five years old were affected by HE allergy. Black children made up 23.4% (95% CI: 13.1–38.4) of those with egg allergies, showing they were overrepresented [28]. This aligns with earlier estimates suggesting that HE allergy affects up to 2.5% of young children [29]. In Australia, a study reported that 8.9% (95% CI, 7.8–10.0) of children had oral food challenge-confirmed allergy to raw eggs [30,31]. In Europe, prevalence rates differ by country; for instance, in Greece, a parent-reported study showed a prevalence of 0.07%, while Germany and the United Kingdom reported rates exceeding 2%. The overall raw incidence of HE allergy by age 2 was 0.84% (95% CI: 0.67–1.03) [32]. Even in China, HE allergy is a very common food allergy among children. The top parent/self-reported allergens in Chinese children up to 5 years are shrimps (1.55%), eggs (1.25%), and crabs (0.99%) [33,34]. In 2012, clinical reports from three Chinese cities (Chongqing, Zhuhai, and Hangzhou) indicated that about 4% of children aged 0–2 years were allergic to HE [35]. Recently, data from the Asia-Pacific Research Network for Anaphylaxis (APRA), which involves China, Thailand, and Singapore, reported that eggs are the most common trigger of anaphylaxis in children aged 3 years and younger [36].

HE allergy in children is mostly resolved by school age. In contrast, only 20% of children with a peanut allergy develop a tolerance by the age of 6 [37]. Other studies report up to 68% resolution of HE allergy until 16 years [38]. While HE allergy is more common in children, it can persist into adulthood or develop later in life. Recent studies have highlighted that HE allergy is relatively rare in adults, with an estimated prevalence of 0.1% [39,40]. However, when present, HE allergy in adults can lead to a significant impairment of health-related quality of life [41]. Children with HE allergy often have other allergic conditions; asthma, eczema, and allergic rhinitis were more prevalent in children with HE allergy compared to those with other food allergies [28]. In the US cohort, over 25% of children with HE allergy experienced severe allergic reactions, and they had higher rates of emergency department visits for allergic reactions compared to children with other food allergies [28].

Recent data indicate that food allergies, including HE allergy, are on the rise among children [42]. A study published in 2024 reported that food allergies in England doubled between 2008 and 2018, with children being the most affected [42]. Significant regional and urban–rural differences in food allergy prevalence were found in a large-scale survey conducted in China with over 70,000 children aged 0–5 years [43]. The prevalence was significantly higher in urban areas (6.37% versus 3.68% in rural areas), and a separate study conducted in Beijing found that the food allergy rate was 2.6% in urban primary school students compared to 0.2% in their rural counterparts [44]. These findings highlight the influence of environmental, dietary patterns, and lifestyle factors in shaping global food allergy epidemiology [45]. Unfortunately, HE allergy remains a significant public health concern, and the increasing trend of food allergies and egg consumption underscores the need for ongoing research to address this growing concern.

## 5. Hen Egg Allergens

The total protein content of HE white was estimated at 110 mg/mL [46]. HE white possesses 23 distinct proteins [47]; however, only 6 allergenic egg proteins from Gallus domesticus (chicken) have been officially recognized in the World Health Organization and International Union of Immunological Societies (WHO/IUIS) Allergen Nomenclature Sub-Committee allergen database [48]. Ovomucoid (Gal d 1), ovalbumin (Gal d 2), ovotransferrin (Gal d 3), and lysozyme (Gal d 4) are known as major allergens in the egg white [49]. Yolk-derived allergens have also been reported, such as α-livetin (Gal d 5) and yolk glycoprotein 42 (Gal d 6) [50]. According to an Italian oral food challenge (OFC) study, serum IgE reactivity to Gal d 1 (20/46 patients, 43.5%), Gal d 2 (24/46 patients, 52.1%), and Gal d 4 (17/46 patients, 36.9%) was more frequent, while fewer patients had IgE reactivity to Gal d 3 (6/46 patients, 13.0%) or Gal d 5 (2/46 patients, 4.3%) [51]. Gal d 6 was first reported as an egg allergen in a 2010 Spanish study when 18% of sera from 27 egg-allergic patients showed IgE binding towards Gal d 6 [52].

With a molecular weight (Mw) of around 28 kDa and 186 amino acid residues, **ovomucoid**, **OVM**, makes up 9.5–11% of egg white. OVM has the highest heat, acid, and enzymatic hydrolysis stabilities of the four, making it the most allergenic [53]. **Ovalbumin**, **OVA**, consisting of 385 amino acid residues with Mw around 45 kDa, makes up more than half of all the proteins in egg whites (54–66%) and provides vital amino acids for the development of the chicken embryo [53,54]. **Ovotransferrin**, **OVT**, has Mw of 77.7 kDa and contains 686 amino acids (12–13% of total protein content) [53]. OVT is a glycoprotein whose peptides have been shown to have antioxidant, antimicrobial, iron transportive, and anti-cancer properties inhibiting tumor growth in vitro [55]. **Lysozyme**, **LYS**, has a Mw of 14.3 kDa and consists of 129 amino acid residues (2.3–4.5% of total protein content). Apart from OVM, other egg white allergens are heat-labile proteins and cooking eggs reduces the likelihood of HE allergy symptoms [53]. **α-livetin**, **LIV**, with 615 amino acid residues and Mw of 69.9 kDa, is highly prevalent in egg yolk, and a similar type of chicken serum albumin is found in bird tissues. Individuals sensitized towards these proteins may also exhibit poultry meat allergy and suffer from bird-egg syndrome [56,57]. **Yolk glycoprotein 42**, **YGP42**, consists of the carboxy-terminal 284 amino acid residues which are cleaved from the primary translation product of vitellogenin-1 (UniProt: P87498) and an apparent molecular weight of 35 kDa. In contrast to chicken serum albumin, YGP42 is thermo-stable [52]. A summary of the overall biochemical features of the egg proteins studied in this article is presented in Table 2. This information will be useful for researchers who are developing their purification flowcharts.

## 6. Isolation of Hen Egg Allergens

Obtaining complete and unaltered protein extracts is the first step in conducting HE allergy research. It is essential to extract the allergens from the matrix quantitatively, reproducibly, and without changing their allergenic potential during the extraction procedure. Furthermore, proteins might interact with the components of the matrix during the extraction, which may alter their immunological behavior [86]. As a result, even with the same food sample, the composition of the matrix may vary depending on the method used to prepare the extract, which may influence the final product’s allergen profile and concentration [87]. Different approaches to extracting egg allergens have been reported in the literature. Six alternative extraction solutions were compared by Hilderbrandt et al. (2008) [88]; extraction with 8 M urea solution yielded the highest protein concentration, but as expected, diminished the allergenic potential of the isolated egg proteins making them inappropriate for immunological studies [55]. After urea, the highest protein content was obtained from the phosphate-buffered saline (pH 7.4) extraction, followed by Tween 20 solution (0.2%), physiological saline (0.15 M), water (pH 8), and acetate buffer (0.1 M, pH 3.8) [88].

Due to their high prices and complicated extraction procedures, most laboratory-scale methods for protein separation are challenging to implement in industrial processes. To date, most of the separation techniques, as opposed to a co-purification procedure, have been developed for isolating just one or two distinct types of egg white proteins. From the standpoint of industrial production, it is more advantageous if numerous proteins in a sample are simultaneously recovered with high purity and yield while maintaining the activity of all target proteins (co-purification) [89]. There are currently two main categories for separating and purifying egg white proteins (EWPs): (1) scale-up methods: ultrafiltration, organic solvent precipitation, salting out, isoelectric precipitation, ion-exchange chromatography, polyethylene glycol precipitation; (2) laboratory-scale methods: electrophoresis, reverse micelles, affinity chromatography, exclusion chromatography, and other techniques [90].

### 6.1. Precipitation of Egg White Allergens

Precipitation is a simple, time- and cost-effective method for separating EWPs. The precipitation of EWPs is achieved using salts (salting-out) and organic solvents, or it can be based on the protein’s isoelectric point [90]. Salting-out is carried out in concentrated salt solutions, followed by desalting to obtain high-purity proteins. For these purposes, it is necessary to use neutral salts to avoid the denaturation of proteins [91]. On the other hand, organic solvents in high concentrations can cause protein denaturation, but also, they are not the best choice for industry in terms of food safety and sustainability [92]. Another important technique relies on the protein’s isoelectric point, where proteins exhibit the lowest solubility. Isoelectric precipitation gives poor results in terms of protein fractionation since EWPs, except for LYS, have similar isoelectric points, but it is beneficial for preparing EWP isolates.

The salting-out of EWPs is usually performed with ammonium sulfate because of its high solubility, low-temperature coefficient, and mild effect on protein structure [91]. Examples of successful isolation via precipitation of EWPs include OVT and OVA. OVT was isolated from EW with various combinations of ammonium sulfate and critic acid in a two-step precipitation scheme followed by ultrafiltration buffer exchange with a yield and purity of over 83% [70]. Gradual ethanol precipitation was another precipitation method used for OVT isolation. Firstly, all the other proteins in egg white were precipitated using 43% ethanol and then OVT was precipitated from the supernatant using 59% ethanol. The negative side of this method is that extreme conditions led to the precipitation of iron-bound OVT (holo-OVT), and iron had to be removed further; the metal-free OVT (apo-OVT) was >80% in purity and around 99% in yield [71]. Polyethylene glycol (PEG) at a final concentration of 15% and pH 6.5 was used to partially separate OVA from other EWPs at 10 °C. OVA-rich supernatant was further purified by isoelectric precipitation at a pH of 4.5 at 4 °C. Obtained OVA had a purity of 95.1%, but the yield was 46.4% [63]. The precipitation of EWPs is a frequent first step in multi-protein fractionation strategies, which will be discussed later in the text.

### 6.2. Co-Purification of Egg White Allergens

Various methods have been explored for the co-purification of EWPs, employing chromatographic techniques and precipitation strategies to achieve high purity and yield (Table 3). Many authors relied on ion exchange chromatography as the main method for isolating EWPs due to its mild separation conditions which should preserve the proteins’ native structure. IEX is a purification method that separates molecules according to their net surface charge. It is based on the interaction between charged molecules in a sample and a column resin with an opposite charge. Cation exchangers typically possess sulfonic (–SO_3_H) or carboxylic (–COOH) acid groups as surface functional groups, whereas anion exchangers are characterized by the presence of tertiary (R_3_N) or quaternary (R_4_N^+^) amine groups. Commercial resins are usually identified by a letter in their name, which indicates a specific functional group (e.g., diethyl aminoethyl (DEAE), carboxymethyl (CM), quaternary ammonium (Q), sulfonate (S), and sulfopropyl (SP)).

Guerin-Dubiard et al. (2005) [64] separated LYS, OVT, OVA, and flavoprotein from mucin-free EW using ion-exchange chromatography columns: S Ceramic Hyper DF (Biosepra, Cergy Saint-Christophe, France) for LYS and OVT, and Q Sepharose Fast Flow (Amersham Biosciences, Uppsala, Sweden) for OVA and flavoprotein. The purity levels of LYS, OVT, OVA, and flavoprotein were 95%, 89%, 91%, and 100%, respectively [64]. When EW was treated with a two-step 100 mM NaCl (pH 6.0)/500 mM NaCl solution rather than water (pH 6.0), ovomucin was isolated with >90% purity [65]. The proteins remaining in the supernatants were further separated by a procedure similar to the one proposed by Guerin-Dubiard et al. (2005) [64], but in reverse order, starting with an anion exchange (Q Sepharose Fast Flow (GE Healthcare, Uppsala, Sweden)) followed by the cation exchange chromatography of the flow-through fraction (SP Sepharose FF (GE Healthcare, Uppsala, Sweden)). OVT, OVA, and flavoprotein were obtained after the anion-exchange chromatography of a 100 mM supernatant. Ovoinhibitor, LYS, and OVT were further isolated from the flow-through fraction. Fractions corresponding to LYS, OVT, and OVA were also obtained after the 500 mM supernatant underwent cation exchange chromatographic separation. The purities of the isolated proteins ranged from 47% to 80%, and the average recovery rate was 71% [66]. Tankrathok et al. (2009) [58] also isolated OVA, OVT, OVM, and LYS via two-step ion exchange chromatography. In the first step, Q Sepharose FF anion exchange chromatography separated LYS and OVA, achieving 87% and 70% purity, respectively. The second step was CM-Toyopearl 650 M (TOSOH Cooporation, Tokyo, Japan) cation exchange chromatography, used to obtain OVT at 80% purity. OVM was precipitated from the partially purified protein fraction with ethanol and trichloroacetic acid to yield 90% pure protein. Despite achieving relatively high purities for LYS, OVA, OVT, and OVM (87%, 70%, 80%, and 90%), the recovery yields were lower (55%, 54%, 21%, and 21%) [58].

There are also examples of the successful utilization of only cation exchange matrices. Two-step membrane cation exchange chromatography (Sartobind S nano (Sartorius Stedim, Göttingen, Germany)) was employed to separate LYS and OVT [67]. This method resulted in a purity of 96% for LYS (yield: 99%, purification factor: 21) and 84% for OVT (yield: 97%, purification factor: 5). Cation exchange CM-Sepharose (GE Healthcare, Uppsala, Sweden) was also used for OVM, OVA, OVT, and LYS, with pretreatment involving ovomucin precipitation and pH adjustment (from 6.0 to 3.8). The final yields were 60.0%, 52.1%, 29.6%, and 90.2%, respectively, with high purity and antigenicity preservation [89].

Quite often the co-purification procedure involves precipitation techniques alongside ion exchange. OVM and OVT were sequentially separated through precipitation with a high concentration of ethanol (61%) and salts (2.5% ammonium sulfate and 2.5% citric acid). OVM was further purified by heating at 65 °C for 20 min to remove the impurities, yielding > 96% for OVM and >92% for OVT, both with purity levels above 88% [59]. The same research group later optimized a continuous isolation process for LYS, OVA, ovomucin, and OVT, employing FPC3500 cation exchange resin (Fisher Scientific, Waltham, MA, USA) for LYS, isoelectric point precipitation for ovomucin, and salting-out methods for OVT and OVA. Desalting via ultrafiltration and heat treatment for OVA ensured high purity, with yields of >98% for OVA and >82% for OVT and LYS [60]. A similar purification strategy was implemented by Ji et al. (2020) [68]; the purity and yield exceeded 90% and 77% for LYS, OVT, OVA, and OVM, while ovomucin achieved 72% purity and 75% yield. Geng et al. (2012) [69] separated ovomucin and most of the OVA by PEG precipitation. Then, Q Sepharose FF anion exchange chromatography was performed to purify LYS, OVT, OVA, and flavoprotein. The purity of products was 91.84%, 94.55%, 96.45% and 88.16%. The recovery yields of ovomucin, LYS, OVT, and OVA were, respectively, 63.59%, 30.10%, 77.75%, 88.64%, and 53.17%. In another study, avidin was removed from the crude egg white extract using a cation exchanger StreamlineTM SP (Pharmacia Biotech, Hong Kong) before the bound LYS was eluted using 5% ammonium carbonate, pH 9.0, containing 1 M NaCl. The additional purification of LYS was achieved by affinity chromatography on dye-linked cellulose beads. OVM and OVA were further purified from the flow-through; OVA was precipitated with 5% trichloroacetic, and OVM was isolated from the supernatant by ethanol precipitation. Yields for LYS, OVM, and OVA were 77%, 94%, and 98%, respectively [61].

Overall, multiple co-purification methods have been successfully implemented to separate EWPs with high purity, but differences in the yield are notable. What all these studies are lacking is the determination of endotoxin in protein preparations which is relevant for immunological studies, and subsequent proof of epitope preservation in serum IgE-binding assays (ELISA, ImmunoCAP) and functional IgE-assays (basophil/mastocyte degranulation assays). Although a co-purification strategy was not implemented, in a study by Jacobsen et al. (2008) [62], OVA, OVT, LYS, and OVM were isolated mainly by ion exchange chromatography with low endotoxin content and high serum IgE binding activity. The authors also stressed that collecting all protein isoforms in the final preparation was not easy, but relevant for further investigation of isoform-specific allergenicity. This particular care is where further egg allergen research should be directed. Another review article offering a detailed overview of the extraction techniques for EWPs explores individual EW protein purification schemes that were not discussed here [90].

### 6.3. Isolation of Egg Yolk Allergens

Research on egg yolk (EY) allergens is scarce compared to EW allergens (Table 3). The characterization and the extraction of the target EY proteins are essential for immunoassay development. LIV, the first reported EY allergen, was extracted from hen EY using phosphate-buffered saline (PBS) with the addition of 0.1% sodium dodecyl sulfate (SDS) and 1 mM dithiothreitol (DTT) (pH 7.2), which enhanced both immunodetection and recovery. LIV also retained the highest solubility and immunoreactivity after heat treatment under neutral pH but showed disintegration and aggregation in acidic and alkaline conditions [72]. The method originally presented by Burley and Vadehra (1979) [73] for the isolation of EY livetins was adapted with minor modifications by Jacobsen and co-authors (2008) [62] for the isolation of LIV. At first, EY granules were precipitated by combining EY with equal volumes of 0.16 M NaCl and ultracentrifuged at 100,000× *g* for 30 min. The granula-free solution (plasma) was mixed with an equal volume of 4 M NaCl and then ultracentrifuged at 100,000× *g* for 20 h. The supernatant was dialyzed against ammonium acetate, pH 6.8, and subjected to anion exchange chromatography using DEAE-Sepharose CL 6B (Pharmacia, Uppsala, Sweden). Fractions containing LIV were further purified by a strong anion exchanger Q-Sepharose FF at pH 8.5. Only after an additional gel filtration step on Superdex 75 (AP Biotech, Stockholm, Sweden) did the protein reach a purity >98%, with a total yield of 10% [62].

YGP42 is the second allergen characterized from EY. In the original study, the protein was isolated from EY via Reverse-Phase High-Performance Liquid Chromatography (RP-HPLC, ACE 5 C4-300 column (Advanced Chromatography Technologies, Aberdeen, Scotland)) [52]. The small quantities of purified protein were of good quality for the subsequent immunological assays with patients’ sera IgE. We did not find any other attempts at YGP42 isolation from EY. However, De Silva et al. (2016) [74] produced a recombinant form of YGP42 in an *Escherichia coli* expression system. Recombinant YGP42 was His tagged to allow an easy isolation procedure via a metal chelating resin (Ni^2+^-nitrilotriacetic (Ni-NTA) resin, Qiagen, Hilden, Germany). The recombinant YGP42 was recognized by patients’ serum IgE, having a binding capacity comparable to that of the native protein [69]. There are also reports of successfully produced recombinant versions of other egg proteins with preserved IgE-binding epitopes such as OVM, OVA, and OVT [75,76]. Recombinant egg proteins can be used in allergen specific immunotherapy (SIT) and diagnostic methods such as skin prick tests (SPT) [77].

**Phosvitin** (**PSV**), a phosphoprotein in egg yolk [78], is not recognized as an egg allergen by the WHO/IUIS Allergen Nomenclature Sub-Committee. While it has been identified as an IgE-binding protein in some individuals with egg allergies, its clinical significance is considered minor compared to other egg proteins [79,81]. Hen EY PSV was first isolated by Mecham and Olcotte in 1949 [82] by precipitation with magnesium sulfate. In their work, Ko et al. (2011) [83] developed a new method for the large-scale ethanol and salt-based isolation of PSV. EY was diluted with water, and the resulting granules, containing PSV, were precipitated. Lipids and phospholipids were removed by 85% ethanol and PSV was extracted using 10% ammonium sulfate or 10% sodium chloride solution at pH 4.0, with further ultrafiltration to remove salts. PSV was recovered using (NH_4_)_2_SO_4_ and NaCl at rates of 72% and 97%, respectively, and at a purity of about 85% [83]. In another study, delipidated granules (via solvent-free method) were dissolved in carbonate–bicarbonate buffer at pH 9.6, resulting in a solution that was used for the separation and purification of PSV by anion exchange chromatography (Q Sepharose FF). As a result, the PSV fraction was isolated with a purity of 92.6% yielding 35.4% [93]. PSV was also isolated from the EY using ultrasonic thermal assistance (UTAE). The sample was heated for 15 min at 80 °C, followed by 10 min of ultrasonic processing at an ultrasonic power of 600 W to effectively extract PSV. In ideal circumstances, the purity and activity were 80% and 98%, respectively [94].

A crucial first step in researching the molecular characteristics of EY allergens is developing effective, high-throughput separation and purification methods. We may examine the relationship between an allergen’s structure and allergenicity more precisely and clearly when high-purity allergens are obtained. Most of the research on the epitopes of EY allergens relies on bioinformatics-based prediction algorithms, which still require serological verification [80,96].

## 7. Isolation of Egg Proteins from Other Avian Species

Most research on avian egg proteins is performed with an extract; therefore, there are not many isolation strategies to report (Table 3). Duck egg albumen is rich in protease inhibitors, similar to hen’s OVM, ovoinhibitor, and ovostatin, which can be used as a protein additive to enhance gelling properties in food products. Therefore, it was of interest to isolate and purify Trypsin inhibitor (TI) from duck egg albumen. TI was purified using ammonium sulfate precipitation at 20–40% of saturation, followed by affinity column chromatography. Trypsin-CNBr-activated Sepharose 4B-trypsin (GE Healthcare, Uppsala, Sweden) was used to perform chromatography. The purity and yield of the product were 111.8-fold and 0.6%, respectively. TI remained stable within the 40–60 °C temperature range and pH range of 7–9. Salt concentrations greater than 5% caused TI’s inhibitory activity to decrease [84]. LYS was another duck egg protein purified from the salted duck egg white, the main by-product in the production process of salted egg yolk, by isoelectric point precipitation (phosphate-buffer solution (0.20 mol/L, pH 6.8)), ultrafiltration, and cation exchange (D152 ion exchange resin, Zhengzhou Ainuo Technology Co., Ltd., Zhengzhou, China). The product showed high purity, with a yield of 0.36% and an enzyme activity of 18,300 U/mg. LYS was fairly stable within a pH of 4–7 and a temperature range of 30–60 °C. The Fe^2+^, Cu^2+^, and Zn^2+^ ions significantly reduced its activity [95]. Suzuki and co-authors (2001) separated OVT, two OVA variants (POA(hi) and POA(lo)), and OVM from pigeon egg white by C4 RP-HPLC (Phenomenex, Torrance, CA), and performed subsequent N-glycan analysis [85].

## 8. Concluding Remarks

Methods for separating and purifying egg allergens should be gentle enough to maintain the structure of the proteins and minimize the risk of endotoxin contamination. These proteins can be utilized in IgE-binding assays, functional cellular assays, and animal models of allergic diseases. The isolation procedure will vary depending on the specific target, so the experimenter should first determine whether the goal is to purify a single egg allergen or multiple allergens. If the aim is to isolate a panel of egg allergens, we recommend employing a co-purification strategy. After separating the egg white from the yolk, mucin should be removed from the egg white before chromatography. Previous studies have successfully used anion and cation exchange resins/columns from different manufacturers. Ion-exchange resins have a high protein binding capacity. These resins can be cleaned and reused; however, caution should be taken regarding the number of times they are reused and the cleaning procedures, as endotoxins can also bind to the resin. If starting with a strong anion exchanger to separate egg white proteins, a buffer of pH 8 can be used to allow ovotransferrin (pI 6.0–7.2) to bind to the matrix next to ovalbumin and ovomucoid. A buffer of pH 6.0 can also be used to reduce ovotransferrin binding so that the collected loosely bound ovotransferrin and unbound lysozyme can be separated by cation exchange. Both approaches were successfully applied in previous reports. The methodology can be reversed; one could start with a strong cation exchanger and later process the unbound proteins by anion exchange, reversing the order of bound and unbound proteins. If further purification is necessary, size exclusion chromatography can be employed; however, authors of previously published studies have typically favored precipitation techniques. While precipitation techniques are useful and cost-effective, they should be limited to milder salting-out methods, which have proven successful for further purifying ovalbumin and ovotransferrin. For optimal separation and easier standardization, we suggest using preparative column chromatography connected to a liquid chromatography system. However, if financial constraints exist, less expensive protein purification options, such as less expensive ion-exchange resins and batch methods, can still be utilized, but this may lead to reduced purity, separation, and quality. The first step in processing egg yolk involves precipitating granules and delipidation. It is encouraged to use solvent-free methods or alternative greener solvents. From the egg yolk supernatant (plasma), α-livetin can be purified, while phosvitin can be extracted and purified from the granules. Both strong and weak anion exchangers have been successfully used for the purification of α-livetin and phosvitin. For phosvitin, salting-out techniques yielded higher quantities, although the overall purity was lower compared to anion exchange chromatography.

The increasing prevalence of hen egg allergy, particularly among infants and children, and the rising consumption of various bird eggs, highlights the need for a deeper understanding of egg allergens. While considerable research has been conducted on the allergenic hen egg white proteins, data on allergens from hen egg yolk and other avian species remain limited. Future research should focus on improving isolation strategies and expanding the scope beyond hen egg white allergens. This will enhance understanding of egg allergy and the bird-egg syndrome. The data obtained from the isolation and purification strategies of hen egg allergens are valuable for future procedures involving allergen isolation from eggs of other avian species. However, caution should be taken, as isoelectric points and molecular weight values may differ between species. Furthermore, standardizing the extraction, purification processes, and recombinant production will ensure consistency in egg allergen quality, thereby improving diagnostic approaches such as providing actual data on the prevalence of egg allergy and better insight into the molecular basis for potential cross-reactivity. Moreover, advances in egg allergen purification techniques and recombinant technology will facilitate the structural analysis of the proteins, which might boost the development of hypoallergenic egg products, improving dietary options for individuals with egg allergies.

## Figures and Tables

**Table 1:** The global daily per capita supply of fresh hen eggs available for human consumption in g/capita/day in 2010 and 2020. Data were retrieved from the FAO Supply Utilization Accounts available at http://www.fao.org/faostat (accessed on 3 and 17 March 2025). The top 30% are marked in each column in red.

		2010	2020	Percent Increase from 2010 to 2020
	Region	g/Capita/Day		
	World	21.56	26.09	21.01%
Africa	Northern Africa	12.21	13.84	13.35%
	Southern Africa	13.06	16.1	23.28%
	Eastern Africa	2.91	3.03	4.12%
	Middle Africa	1.01	1.07	5.94%
	Western Africa	6.45	5.67	−12.09%
Americas	Northern America	37.92	43.4	14.45%
	Central America	42.94	48.3	12.48%
	Caribbean	14.77	19.97	35.21%
	South America	22.46	31.8	41.59%
Asia	Central Asia	13.4	21.68	61.79%
	Eastern Asia	41.13	48.97	19.06%
	Southern Asia	6.67	11.04	65.52%
	Southeastern Asia	12.17	26.68	119.23%
	Western Asia	15.7	20.69	31.78%
Europe	Eastern Europe	38.29	39.88	4.15%
	Northern Europe	26.12	28.01	7.24%
	Southern Europe	30.4	31.6	3.95%
	Western Europe	33.46	37.59	12.34%
Oceania	Australia and New Zealand	18.45	21.96	19.02%
	Melanesia	2.98	3.12	4.70%
	Micronesia	2.89	11.78	307.61%
	Polynesia	18.46	19.41	5.15%

**Table 2:** Biochemical characteristics of egg allergens registered in the WHO/IUIS allergen database and egg proteins from other species investigated in the study.

Protein	Apparent Molecular Weight (kDa)	Measured Isoelectric Point (pI)	Egg White (EW) or Yolk (EY)	Characteristics	References
*HEN*					
**Ovomucoid (OVM)** Gal d 1 (WHO/IUIS) P01005 (UniProt)	28–37	3.7–4.5	EW	Glycoprotein, trypsin inhibitor, high heat, acid, and enzymatic hydrolysis stability.	[18,53,58,59,60,61,62]
**Ovalbumin (OVA)** Gal d 2 (WHO/IUIS) P01012 (UniProt)	41–46	4.5–4.8	EW	Globular phosphoglycoprotein, the major EW protein, a heat-labile, serin protease inhibitor.	[18,27,53,54,55,58,59,60,61,62,63,64,65,66,67,68,69]
**Ovotransferrin (OVT)** Gal d 3 (WHO/IUIS) P02789 (UniProt)	75–79	6.0–7.2	EW	Glycoprotein, transports iron, has antimicrobial properties, and is heat labile.	[18,53,58,59,60,62,63,64,65,66,67,70,71]
**Lysozyme (LYS)** Gal d 4 (WHO/IUIS) P00698 (UniProt)	14.3	10.7	EW	Antimicrobial (hydrolyzes bacterial cell walls) and heat labile.	[18,53,58,59,60,62,64,65,66,67]
**α-livetin (LIV)** Gal d 5 (WHO/IUIS) P19121 (UniProt)	65–70	4.6–4.8	EY	Highly prevalent in EY, similar to chicken serum albumin in bird tissues, and is heat labile.	[56,57,62,72,73,74]
**Yolk glycoprotein 42 (YGP42)** Gal d 6 (WHO/IUIS)	35, 42	5.88 theoretical	EY	Cleaved from the primary translation product of vitellogenin-1 (UniProt: P87498), heat stable.	[53,74,75,76,77,78,79,80]
**Phosvitin (PSV)**	35–45	4	EY	Phosphoprotein, heat stable. IgE binding detected, minor clinical significance.	[73,80,81,82,83]
*DUCK*					
**OVA**	40–48		EW	Similar to hen egg OVA.	[23]
**Trypsin inhibitor (TI)**	43		EW	Serine protease inhibitor, stable within 40–60 °C at pH of 7–9.	[84]
**LYS**	14	>10	EW	Thermostable between 30 and 60 °C at pH of 4–7.	[78]
*PIGEON*					
**OVM**	45		EW		[85]
**OVA**	49–53		EW		[85]
**OVT**	76		EW		[85]

**Table 3:** Selected methods for the isolation of hen egg allergens and other avian species egg proteins.

Hen Egg						Other Eggs						Details	References
Egg White			Egg Yolk			Duck Egg White			Pigeon Egg White				
OVM	OVA	OVT	LYS	LIV	YGP42	PSV	TI	LYS	OVA	OVT	OVM		
	✓											(1) PEG, (2) pH 4.5 precipitation. Yield: 46.4%, purity: >95.1%.	[63]
		✓										AS/citric acid precipitation. Yield and purity: ≥83%.	[70]
		✓										Ethanol precipitation. Yield: 99%, purity: >80%.	[71]
✓		✓										(1) Ethanol, (2) AS/citric acid precipitation. (3) Heating (for OVM). Yield: OVM > 96%, OVT > 92%. Purity: OVM and OVT > 88%.	[59]
		✓	✓									Two-step membrane cation exchange/Sartobind S nano. Yield: LYS 99%, OVT 97%. Purity: LYS 96%, OVT 84%.	[67]
✓	✓		✓									(1) LYS—Cation exchange/Streamline^TM^ SP, affinity chromatography/Cibacron Blue F3GA. (2) OVA: TCA precipitation, OVM: ethanol precipitation. Yield: OVM 94%, OVA 98%, LYS 77%.	[61]
	✓	✓	✓									(1) Mucin removal. (2) LYS, OVT: cation exchange/S Ceramic Hyper DF. (3) OVA: anion exchange/Q Sepharose FF. Yield: LYS 100%, OVT 78%, OVA 50%. Purity: LYS 95%, OVT 89%, OVA 91%.	[64]
	✓	✓	✓									(1) Mucin extraction. (2) OVA, OVT: anion exchange/Q Sepharose FF. (3) OVT, LYS: cation exchange/SP Sepharose FF. Purities ranged from 47 to 80%, average yield 71%.	[65,66]
	✓	✓	✓									(1) LYS: cation exchange/FPC3500. (2) OVA, OVT: AS/citric acid precipitation. Yield: OVA > 98%, OVT and LYS > 82%. Purity > 90%.	[60]
	✓	✓	✓									(1) Mucin removal via PEG precipitation. (2) OVA, OVT, LYS—Anion exchange/Q Sepharose FF. Yield: OVA 53.17%, LYS 30.10%, OVT 77.75%. Purity: OVA 88.16%, LYS 94.55%, OVT 96.45%.	[69]
✓	✓	✓	✓									(1) Mucin removal. (2) OVA, LYS: anion exchange/Q Sepharose FF. (3) OVM, OVT: cation exchange/Toyopearl CM-650 M. (4) OVM: ethanol/TCA precipitation. Yield: LYS 55%, OVA 54%, OVT 21%, OVM 21%. Purity: LYS 87%, OVA 70%, OVT 80%, OVM 90%.	[58]
✓	✓	✓	✓									(1) Mucin removal. (2) OVM, OVA, OVT, LYS: Cation exchange/CM-Sepharose. Yield: OVM 60%, OVA 52.1%, OVT 29.6%, LYS 90.2%.	[89]
				✓								(1) Granula removal. (2) Anion exchange/DEAE-Sepharose CL 6B. (3) Anion exchange/Q Sepharose FF. (4) Gel filtration/Superdex 75. Yield 10%, purity > 98%	[62]
					✓							RP-HPLC/ACE 5 C4-300, good quality for immunoassays.	[53]
					✓							His-tagged recombinant protein. Affinity chromatography/NI-NTA. IgE reactive.	[74]
						✓						(1) Ethanol delipidation. (2) Salt-based isolation (NaCl or AS). Yield 72% (AS) and 97% (NaCl).	[83]
						✓						(1) Solvent-free delipidation. (2) Anion exchange chromatography/Q Sepharose FF. Yield 35.4%, purity 92.6%.	[93]
						✓						(1) Heating, 80°, 15 min. (2) Ultrasonic processing 600 W, 15 min. Purity 80%.	[94]
							✓					(1) AS precipitation. (2) Affinity chromatography/Trypsin-CNBr-activated Sepharose 4B. Yield 0.6%, 111.8-fold increase in purity.	[84]
								✓				(1) Isoelectric point precipitation (pH 6.8). (2) Cation exchange/D152 resin. Yield 0.36%.	[95]
									✓	✓	✓	RP-HPLC/C4 column. Suitable for mass spectrometry.	[85]

Abbreviations: OVM—ovomucoid, OVA—ovalbumin, OVT—ovotransferrin, LYS—lysozyme, LIV—α-livetin, YGP42—yolk glycoprotein 42, TI—trypsin inhibitor, AS—ammonium sulfate, PEG—polyethylene glycol, TCA—trichloroacetic acid, RP-HPLC—reversed-phase high-performance liquid chromatography. Chromatographic methods are presented as method/commercial column name or method/commercial resin name; manufacturers are listed in the main text.
